# Towards quantitative perfusion MRI of the lung in COPD: The problem of short-term repeatability

**DOI:** 10.1371/journal.pone.0208587

**Published:** 2018-12-10

**Authors:** Alvard Ter-Karapetyan, Simon M. F. Triphan, Bertram J. Jobst, Angela F. Anjorin, Julia Ley-Zaporozhan, Sebastian Ley, Oliver Sedlaczek, Jürgen Biederer, Hans-Ulrich Kauczor, Peter M. Jakob, Mark O. Wielpütz

**Affiliations:** 1 Department of Diagnostic & Interventional Radiology, University Hospital of Heidelberg, Heidelberg, Germany; 2 Translational Lung Research Center Heidelberg (TLRC), Member of the German Lung Research Center (DZL), Heidelberg, Germany; 3 Department of Diagnostic and Interventional Radiology with Nuclear Medicine, Thoraxklinik at University of Heidelberg, Heidelberg, Germany; 4 Research Center Magnetic Resonance Bavaria (MRB), Würzburg, Germany; 5 Institute for Clinical Radiology, Ludwig-Maximilians-University Hospital Munich, Munich, Germany; 6 Diagnostic & Interventional Radiology, Chirurgisches Klinikum München Süd, Munich, Germany; 7 Radiologie Darmstadt, Department of Radiology, County Hospital Gross-Gerau, Gross-Gerau, Germany; 8 Department of Experimental Physics Julius-Maximilians Universität, Würzburg, Germany; University of Wisconsin, UNITED STATES

## Abstract

**Purpose:**

4D perfusion magnetic resonance imaging (MRI) with intravenous injection of contrast agent allows for a radiation-free assessment of regional lung function. It is therefore a valuable method to monitor response to treatment in patients with chronic obstructive pulmonary disease (COPD). This study was designed to evaluate its potential for monitoring short-term response to hyperoxia in COPD patients.

**Materials and methods:**

19 prospectively enrolled COPD patients (median age 66y) underwent paired dynamic contrast-enhanced 4D perfusion MRI within 35min, first breathing 100% oxygen (injection 1, O_2_) and then room air (injection 2, RA), which was repeated on two consecutive days (day 1 and 2). Post-processing software was employed to calculate mean transit time (MTT), pulmonary blood volume (PBV) and pulmonary blood flow (PBF), based on the indicator dilution theory, for the automatically segmented whole lung and 12 regions of equal volume.

**Results:**

Comparing O_2_ with RA conditions, PBF and PBV were found to be significantly lower at O_2_, consistently on both days (p<10–8). Comparing day 2 to day 1, MTT was shorter by 0.59±0.63 s (p<10–8), PBF was higher by 22±80 ml/min/100ml (p<3·10–4), and PBV tended to be lower by 0.2±7.2 ml/100ml (p = 0.159) at both, RA and O_2,_ conditions.

**Conclusion:**

The second injection (RA) yielded higher PBF and PBV, which apparently contradicts the established hypothesis that hyperoxia increases lung perfusion. Quantification of 4D perfusion MRI by current software approaches may thus be limited by residual circulating contrast agent in the short-term and even the next day.

## Introduction

For lung diseases like chronic obstructive pulmonary disease (COPD), cystic fibrosis and asthma, function parameters available through spirometry are well-established in clinical routine. However, these global tests do not reflect regional distribution of lung disease or treatment response on a lobar or segmental level [1–4]. Thus, non-invasive imaging methods dedicated to collecting regional information on lung structure and function are important research tools in the field. In contrast to computed tomography (CT), proton magnetic resonance imaging (MRI) can be repeated arbitrarily due to lack of radiation exposure. In cystic fibrosis, this has led to the implementation of morpho-functional MRI into clinical routine monitoring of disease progression, and for clinical trials in specialized centers [5–7]. Dynamic contrast-enhanced four-dimensional (4D) perfusion MRI has been shown to be useful for visualizing lung function deficits in the form of perfusion abnormalities: Due to the mechanism of hypoxic vasoconstriction caused by airway obstruction [6, 8], perfusion parameters can be used as a biomarker for therapy response [5, 7]. Subsequently, it was demonstrated that visual scoring of 4D perfusion has low inter-reader variability in cystic fibrosis and COPD patients [9, 10], low short-term variation within 24 h in clinically stable COPD patients [10], and detects therapy response within 1 month in pediatric and adult cystic fibrosis patients [5, 7]. For larger clinical trials, it is however desirable to both minimize user-interaction and thus possible observer bias, and to produce comparable quantitative values reflecting lung function, i.e. regional lung perfusion. Typically, visual scoring focuses on signal intensities on subtracted perfusion maps. To exploit the entirety of available data from 4D perfusion MRI, including the time component of the contrast agent bolus passage through the lung parenchyma, requires software analysis. The indicator dilution theory has been used to calculate indices of lung perfusion such as mean transit time (MTT), pulmonary blood flow (PBF), and pulmonary blood volume (PBV) by dedicated post-processing software [11–13]. As further improvement, automatic segmentation of the lungs based on gradient echo images has been shown to be robust and may be transferred to 4D perfusion datasets to calculate aforesaid perfusion indices for the whole and segmented lung regions [14]. In order to further evaluate the methodology and to simulate effects observed in a pharmacological trial, iatrogenic hyperoxia may serve as a valuable model to induce lung perfusion changes. The expected effect is to increase lung perfusion in COPD, exploiting the effect of hypoxic pulmonary vasoconstriction [15, 16]. Therefore, the aim of the present study was to assess the short-term response to hyperoxia in 19 COPD patients with repeated 4D perfusion MRI including quantitative post-processing to derive changes in MTT, PBF and PBV. Further, the intra-individual reproducibility of this response within 24 h was studied, with the intention to establish this experimental setup of repeated 4D perfusion MRI for subsequent interventional trials.

## Materials and methods

### Ethics statement

The study was carried out as part of a prospective trial (ASCONET, German Clinical Trials Register number DRKS00005072) approved by the institutional ethics committee, and conducted according to the recommendations of the institutional review board. The work was carried out in accordance with The Code of Ethics of the World Medical Association (Declaration of Helsinki). Prior to participation in the study, all subjects gave their written informed consent.

### Study design and patient characteristics

19 patients, aged 49 to 79 years (median 66 years) diagnosed with COPD and different disease severity according to the GOLD criteria were enrolled [17]. The cohort included n = 10 patients with mild COPD (GOLD stages I-II) recruited from an outpatient clinic, and n = 9 patients with severe COPD (GOLD stages III-IV) from a specialized chest clinic (Table 1). To ensure patient safety, the glomerular filtration rate (GFR) was determined within 14 days prior to MRI, excluding patients with a GFR below 40 ml / min.

**Table 1:** Patient characteristics.

	GOLD I & II	GOLD III & IV	Total
**Subjects n**	10	9	19
**Male / Female (n)**	9/1	8/1	18/2
**Minimum age (y)**	49	60	49
**Maximum age (y)**	77	79	79
**Median age (y)**	59 (Q1:55; Q3:70)	69 (Q1:63; Q3:74)	66 (Q1:59; Q3:74)

### Magnetic resonance perfusion imaging

The MRI protocol was specifically tailored to the demands of structural and functional alterations of the pulmonary parenchyma, vasculature and airways using a clinical 1.5T scanner (Magnetom Avanto, Siemens Medical Solutions, Erlangen, Germany) and was performed including morphological and functional sequences as previously described [10, 18]. Dynamic contrast enhanced first pass 4D perfusion MRI was based on a gradient echo sequence with view sharing and stochastic trajectories (TWIST) at high temporal resolution (1.47 s/volume). To minimize signal loss due to the short T2* times in the lungs, images were acquired at TE = 630μs, using an asymmetrical acquisition scheme. In inspiratory breath-hold the contrast bolus was injected by a power injector (0.05 mmol/kg Gd-DTPA, Magnevist, Bayer Schering Pharma AG, Berlin, Germany) at a rate of 5 ml/s followed by a bolus chaser of 30 ml NaCl. Each acquisition provided 24 consecutive 3D images, covering a time span of at most 37 s. To ensure the availability of a baseline signal, contrast agent was injected 5 s after the start of the measurement, which resulted in between 5 and 8 images without enhancement in the lungs, depending on the individual patients’ circulation. Patients were advised to hold their breath in inspiration as long as possible and then breathe shallowly.

The imaging protocol was identical on both days: Morphological 3D GRE sequences (VIBE) in coronal and transversal plane were performed first. Then, 100% O_2_ was supplemented using a standard clinical facial mask and, after 5 min to ensure complete wash-in, the first 4D perfusion MRI was acquired (‘injection 1’, O_2_). Afterwards, the breathing mask was removed and return to normoxic conditions was ensured over 5 min. Finally, a room-air (‘injection 2’, RA) measurement (equalling 21% O_2_) was acquired as a second 4D perfusion MRI 32±12 min after the first contrast agent injection.

### Quantitative post-processing of 4D perfusion MRI

All datasets were analysed using dedicated quantification software (PulmoMR, Fraunhofer Mevis, Bremen, Germany) employed as previously described [14], including automatic segmentation of the lung. Segmentation results were inspected and corrected manually by two senior radiologists if necessary. Since the resolution of the morphological MR images is insufficient to detect fissures, a geometric partitioning of the lungs is used, which generates for each lung six regions (upper, middle and lower, each separated in front and back) with uniform volume to allow for regional analysis of lung perfusion parameters.

As described previously, lung volume and region volume, as well as median MTT, PBF and PBV were calculated based on the indicator dilution theory for each lung region and for the whole lung [11–13]: The time dependent signal in the pulmonary trunk, which is detected automatically [19], is used as arterial input function (AIF). The tissue response function in each voxel is then determined using a deconvolution with the AIF [20] and MTT, PBF and PBV are taken directly from the shape and amplitude of this function.

As a measure of the intra-patient inhomogeneity of the perfusion parameters, the median absolute difference (MAD) of each parameter in each lung region compared to the overall median in the entire lungs was calculated as well. Perfusion inhomogeneity was further examined by comparing the ventral and dorsal regions as well as comparing the apical and basal regions.

To test the influence large vessels, voxels associated with them were detected [21] and filtered out, resulting in a second dataset.

### Statistical analysis

All data were recorded and analyzed using SciPy [22]. Data are presented as mean ± standard deviation. The differences in parameter values between both injection 1 (O_2_) and injection 2 (RA) as well as between day 1 and day 2 were compared using the method of Bland and Altman [23] and Wilcoxon signed-rank tests. A p-value < 0.05 was considered statistically significant.

## Results

### Reproducibility of automatically generated parameter maps

#### Performance of the automated segmentation

**Fig 1:**
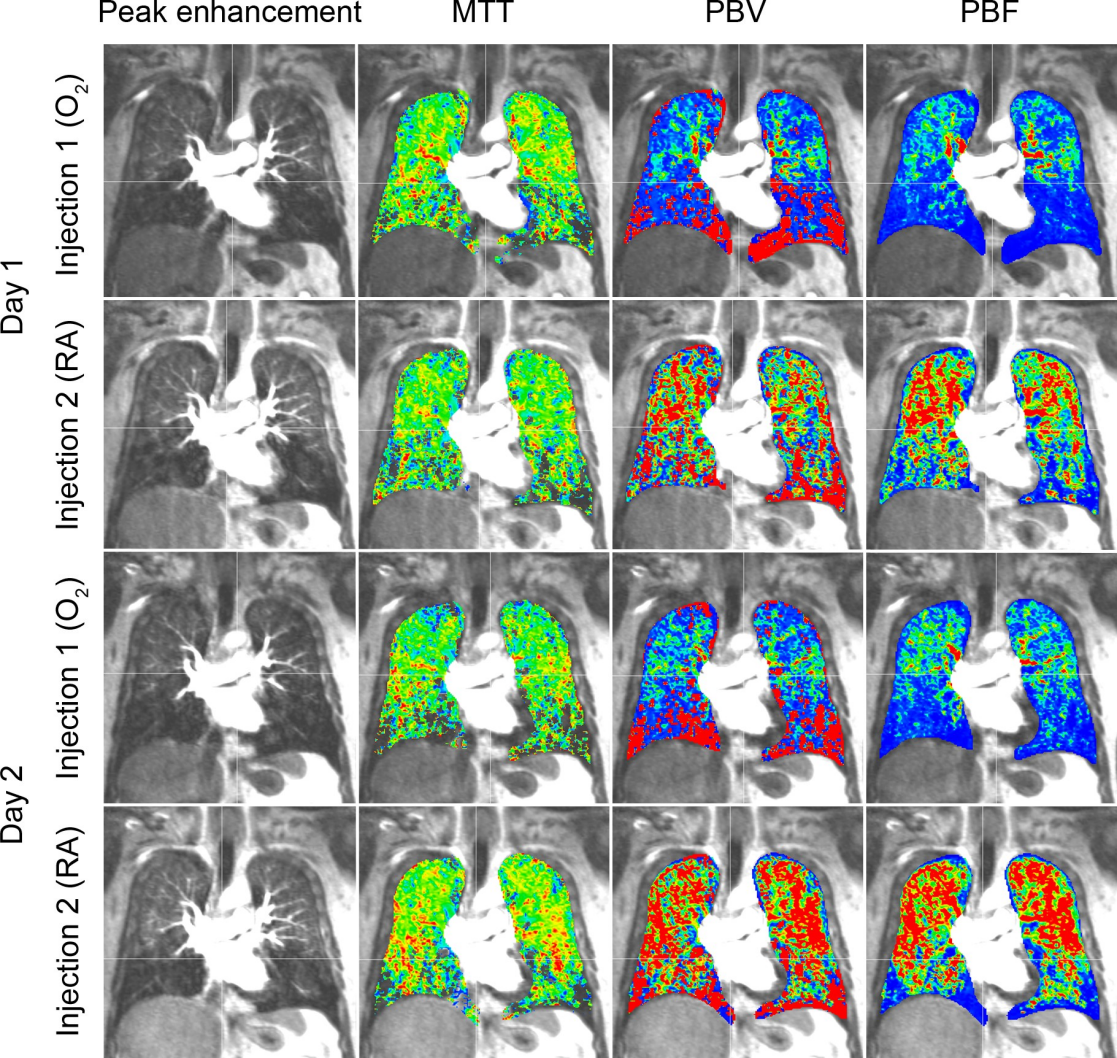
Representative examples of parameter maps. Color-coded parameter maps shown for day 1 and day 2 as well as for injection 1 (O_2_) and injection 2 (room air, RA). MTT = mean transit time, PBF = pulmonary blood flow, PBV = pulmonary blood volume.

Manual correction of the automatically generated segmentation maps was necessary in 2 out of 19 patients on both days (10.5%), which took 10 minutes on average as described previously [14]. Parameter maps could be calculated for all 19 patients (100%). Segmented lung volumes did not differ between injection 1 and 2 nor between day 1 and 2 (Figure A in S1 File). A representative example of MTT, PBF and PBV maps for all measurements in one patient is shown in Fig 1.

#### Apparent influence of oxygen on lung perfusion in COPD

**Fig 2:**
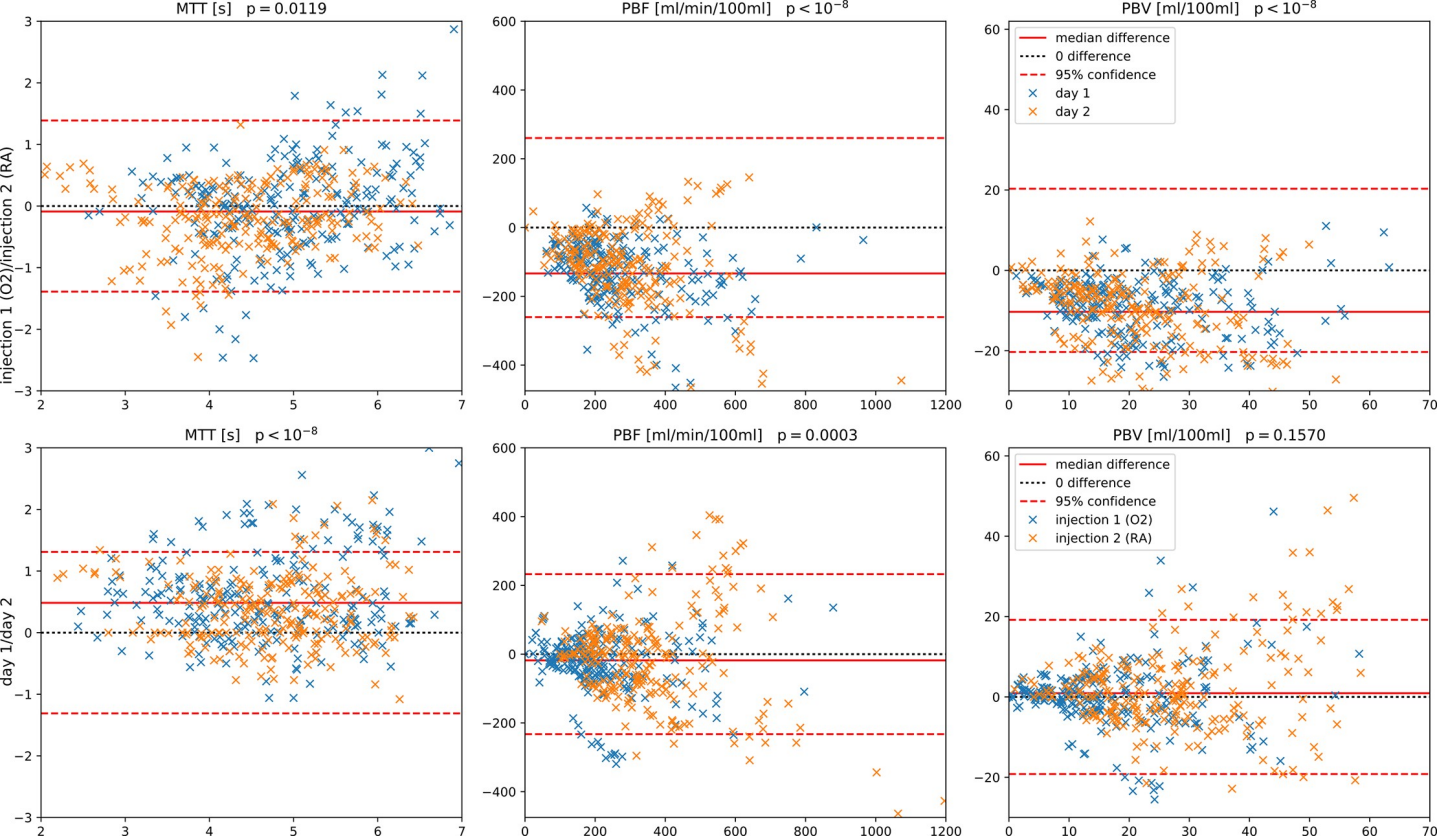
Bland-Altman plots for regional reproducibility. Each lung was divided into 12 regions. Comparisons for injection 1 (O_2_) vs. injection 2 (room air) combining both days are given in the top row (data points for each day are drawn in different colors for better visualization), and comparisons for day 1 vs. day 2 combining both injections are given in the bottom row (in different colors, analogous to the above). MTT = mean transit time, PBF = pulmonary blood flow, PBV = pulmonary blood volume.

The comparison between measurements at injection 1 (O_2_) and injection 2 (RA) conditions for all lung regions and patients on day 1 and 2 is summarized in Fig 2. PBF and PBV were found to be significantly higher when breathing room air (injection 2) compared to hyperoxia (injection 1) (Fig 2 and Table 2), which was a consistent result on both days (p<10–8). Importantly, the AIF was not different between both injections (Table 2).

**Table 2:** Whole-lung perfusion indices.

	Day 1		Day 2		Both days injection 1 vs. 2	Both injections Day 1 vs. 2
	Injection 1 (O_2_)	Injection 2 (RA)	Injection 1 (O_2_)	Injection 2 (RA)	p	p
**MTT (s)**	5.96 ± 0.90	4.94±0.78	4.39 ± 0.87	4.56 ± 0.79	0.012	<10–8
PBF (ml/min * 100ml)	198 ± 116	337±157	220 ± 119	351 ± 163	<10–8	3·10–4
**PBV (ml/100ml)**	16.0 ± 9.7	27±13	15.8 ± 9.6	26 ± 11	<10–8	0.159
**AIF (arb. units)**	1319 ± 356	1312 ± 352	1230 ± 421	1220 ± 417	0.89	0.24

MTT = mean transit time, PBF = pulmonary blood flow, PBV = pulmonary blood volume, AIF = signal integral over the arterial input function. Data given as mean ± SD.

#### Apparent short-term reproducibility of perfusion MRI in COPD

In analogy to the comparison between injection 1 and 2, perfusion parameters were compared for day 1 and 2 considering both injections and all lung regions. Systematic differences were found as shown in Fig 2: On average, MTT was 0.59 ± 0.63 s shorter (p<10–8) on day 2, PBV was lower by 0.2 ±7.2 ml / 100 ml (p = 0.159) and PBF was higher by 22 ± 80 ml / min / 100 ml (p<0.001). Note that the relative difference in MTT is much larger than in PBF. These differences found between both injections as well as both days were found to be independent of disease status when comparing COPD GOLD I and II vs. GOLD III and IV stages, with the sole exception of MTT: When comparing the measurements during injection 1 and 2, MTT was longer at injection 2 (RA) for GOLD stages 3 and 4 (p<0.002), but not for GOLD 1 and 2 (p = 0.69). Again, calculated AIF was not different between both days (Table 2). The above statistical tests repeated on perfusion parameters derived from lung segmentations with large vessels filtered out yielded overall only slightly more significant results. Since this has only minimal effect on the values found at the cost of additional complexity, these results are given in Figure B in S1 File.

#### Regional heterogeneity of perfusion abnormalities in COPD

As a measure of regional inhomogeneity, the MAD for each parameter across the 12 regions per lung was calculated for each individual, and averages across the population are given in Table 3. MAD of PBF and PBV were significantly higher at injection 2 compared to injection 1 consistently on both days (p<10–8), whereas MAD of MTT was smaller at injection 2 only on day 1. When comparing day 1 vs. day 2, we did not detect significant differences in MAD, with the exception of PBF.

**Table 3:** Regional heterogeneity.

	Day 1		Day 2		Both days injection 1 vs. 2	Both injections Day 1 vs. 2
	Injection 1 (O_2_)	Injection 2 (RA)	Injection 1 (O_2_)	Injection 2 (RA)	p	p
**MTT (s), MAD**	0.33 ± 0.17	0.24 ± 0.08	0.31 ± 0.13	0.31 ± 0.15	3·10–3	0.43
PBF (ml/min * 100ml), MAD	48 ± 24	64 ± 24	54 ± 27	67 ± 27	<10–8	4·10–4
**PBV (ml/100ml), MAD**	4.4 ± 1.9	6.2 ± 2.6	4.6 ± 2.5	6.1 ± 2.8	<10–8	0.20

MTT = mean transit time, PBF = pulmonary blood flow, PBV = pulmonary blood volume. The median of the absolute difference (MAD) of the median in each region from the overall median in the lung represents intra-patient heterogeneity, and data are aggregated as mean ± SD for each parameter for all patients.

**Fig 3:**
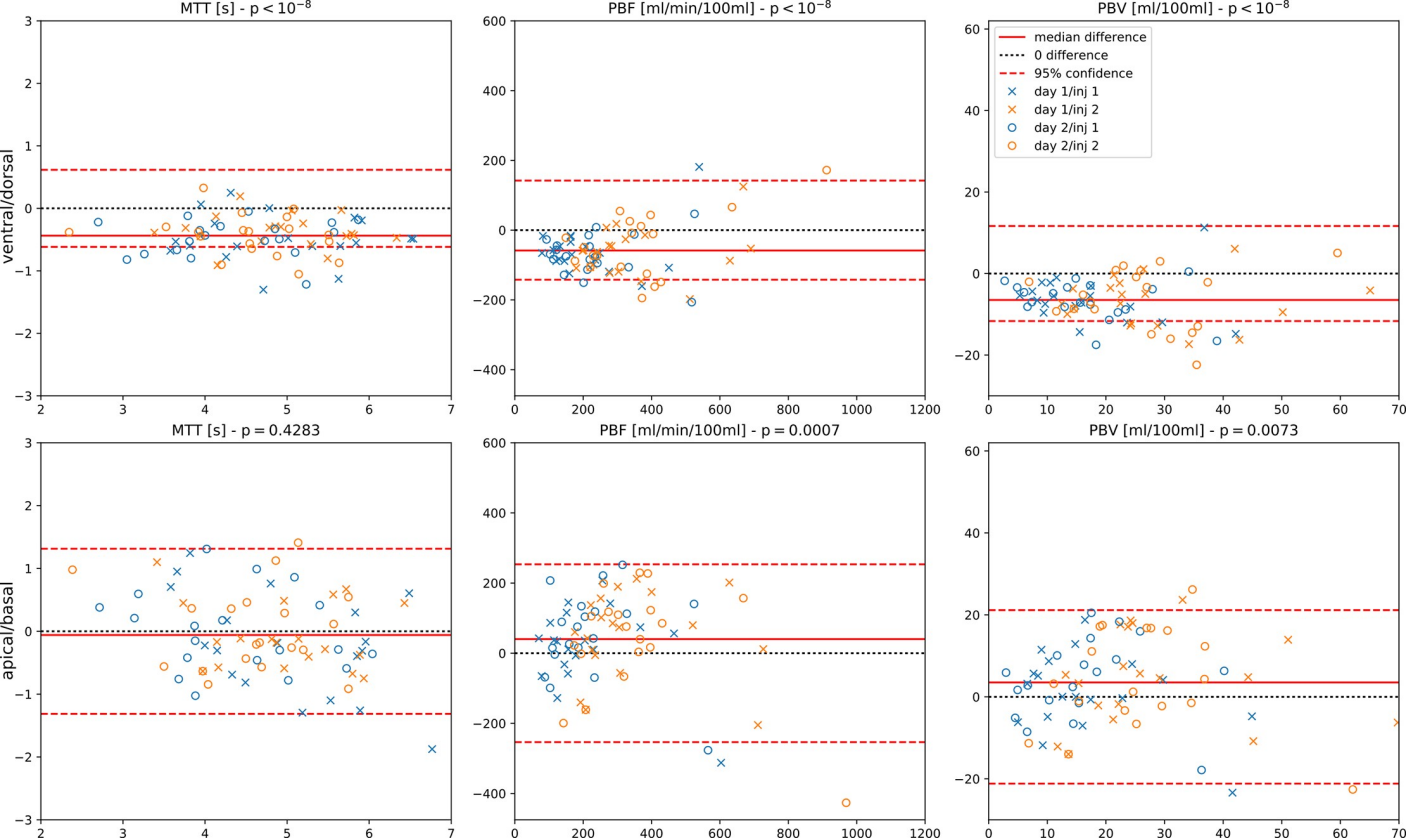
Bland-Altman illustrating regional perfusion gradients. The top row shows the influence of gravity, comparing perfusion parameters averaged over all dorsal regions with ventral regions. The bottom row shows an equivalent comparison of apical and basal regions (the regions in the middle are ignored here).

Fig 3 shows comparisons for the perfusion parameters between the dorsal and ventral lung regions, illustrating the effect of gravity on perfusion, and between apical and basal lung regions, illustrating gravity-independent variability. When comparing the mean values for these regions, the dorsal/ventral difference in MTT, PBF and PBV was significant at p<0.01 for individual measurements and at p<10–5 for all measurements, demonstrating higher perfusion in the gravity-dependent lung regions. Apical to basal differences were not significant individually, but significant at p<0.01 when considering measurements from all days and injections together.

## Discussion

Considering the goal to use 4D perfusion MRI as a robust and reproducible endpoint in interventional trails and to minimize observer bias by using fully automated regional analysis of these datasets, software approaches are essential. In previous studies, we could show that image quality and visual scoring of MRI findings including perfusion abnormalities is highly reproducible in COPD patients [10]. In the present study, we sought to examine whether small changes in lung function in COPD induced by hyperoxia can be detected using 4D perfusion MRI quantification using a repeat measurement after a short time interval, and whether the results can be reproduced within 24 h.

In a previous study Ley-Zaporozhan et al. assessed the reproducibility of the quantification of 4D perfusion MRI within one day in 14 healthy volunteers [24]. They used manually selected regions of interest (ROI) to calculate MTT, PBF and PBV for 2 observers, and demonstrated a high intra-observer reproducibility with non-significant differences between day 1 and day 2 for MTT and PBV, but significant for PBF. However, calculated parameters were also highly dependent on the observer, showing the need for automated quantification to rule out variability related to manual interaction [24]. In another precursor study, a similar perfusion quantification experiment under room air and 100% O_2_ conditions was performed in 10 healthy volunteers, in which a significant increase in PBF under hyperoxia was found [25]. Notably, in that study room-air was the first condition examined with 4D perfusion MRI, with hyperoxia being second. Though we examined COPD patients in our present study, we expected to also detect an increase in pulmonary perfusion parameters (PBV and PBF), as had been previously described employing electrical impedance tomography in COPD patients [16]. In our study hyperoxia was used to simulate the effects of therapy and would ideally provide measurable effects. Unexpectedly, the observed perfusion parameters were apparently reduced in comparison to the state while breathing room-air: We found a significant increase of PBF when measuring room-air conditions second (Fig 2, Table 2). Furthermore, the measurements shown in the present study demonstrate significant systematic biases in both the repeat measurements on the same day as well as those on consecutive days, except for PBV, after 24h (Fig 2 and Table 2).

One possible explanation of this offset is residual circulating contrast agent accumulating with repeat injections. The time dependent signal in the pulmonary trunk is used as AIF and the tissue response function in each voxel in the segmented lung is used to calculate and MTT, PBF and PBV by a deconvolution of the AIF. In principle, the computer-aided image evaluation software considers a baseline signal in the vasculature as well as in the lung tissue prior to, and the signal changes subsequently introduced by the contrast agent injection for parameter calculation. However, due to the non-linear relationship of MRI signal enhancement and contrast agent concentration [13], the highest signal amplitudes will be dampened in the actual measurement. Since this means that higher signals will be affected less by residual contrast agent, the peak of the measured AIF may also be dampened due to non-linearity with subsequent injections. This was actually demonstrated by our measurements showing no statistically significant differences in calculated AIF between both injections and days (Table 2). In turn, as contrast agent will be dispersed in lung tissue and thus far less concentrated, lung signal is more susceptible to residual contrast agent at following injections, which may then lead to an overestimation of the tissue response function after deconvolution. As PBF is determined from the height of the tissue response function, this would explain an erroneously large PBF and, by extension, PBV in the second injection measurements. As a consequence, the effects of short-term influences like the administration of oxygen in this study, may be obscured by the biases introduced by residual contrast, which cannot be compensated for by the currently available experimental setup and software. More recently, corrections for the non-linearity of AIF signal have been proposed and may reduce this bias contribution [26].

One would expect that contrast injections on consecutive days should be unproblematic, but considering in detail the Gd-DTPA application at a dose of 0.1 mmol/kg (for both injections together) employed in the present study, the mean serum elimination half-life would be 1.6 ± 0.1h for healthy subjects with normal renal function [27]. Thus, one would assume that after 24 h no residual effect is measurable, as less than 0.005% circulating contrast agent should remain. It has however been shown that patients with COPD commonly suffer from chronic renal impairment even at normal serum creatinine levels [28]. While a low GFR was an exclusion criterion, the elimination half-life of Gd-DTPA has been shown to increase to 4.2 ± 2.0 h at clearance rates between 30 ml / min and 60 ml / min [27]. As renal function was monitored in this study purely for patient safety reasons, GFRs were not recorded as part of data acquisition and could not be correlated directly with the MRI results. Nevertheless, careful consideration of the comorbidities of the pathology in question appears advisable when proposing repeat measurements for therapy monitoring. Of note, in the measurements on the same day, residual contrast agent would be expected even at optimal kidney function.

Further, we suspect that another effect might have influenced the repeat measurements. As shown recently in interstitial lung disease [29], active inflammation may cause capillary leakage and Gd-extravasation into the pulmonary interstitium after the first injection, which may also persist over 24h. This change in compartments with immobilization of Gd-based contrast agent only affects the extra- but not the intravascular signal including the pulmonary arteries, and thus may strongly influence the measurement of quantitative perfusion parameters.

An influence of contrast agent on segmentation is unlikely, as gradient echo sequences acquired before the first injection are used to determine lung borders and regions for both perfusion measurements. Further we show, that segmented volumes were not different between injections and days (Figure A in S1 File). At most, patients may have moved between perfusion measurements, leading to a mismatch of lung regions between both. However, this would only introduce a random error in a small number of voxels and cannot explain the systematic bias between measurements discussed above.

Dynamic contrast enhanced perfusion measurements are additionally vulnerable to multiple distortions: While in expiration, both proton density is higher and T2* is longer, resulting in increased signal, COPD patients in our experience already have difficulties holding their breath in inspiration. Accordingly, all experiments in this study had to be performed in inspiratory breath-holds due to the time required for a full pass-through of contrast agent through the lungs. As mentioned above, lung segmentations showed no statistically significant differences between the measurements on consecutive days or the same day (Figure A in S1 File).

Local functional measurements like the quantification of lung perfusion attempted here have the potential to not only reveal global changes in biomarkers, but also to observe the regional inhomogeneity as well. As shown in Fig 3, the increase of blood volume due to gravity is clearly visible in all individual measurements shown here. As COPD patients are expected to have areas with different degrees of impairment in their lungs, it is not surprising to find an intra-patient variability larger than the difference seen here from one day to the contrast agent injection on the next (Table 3, Fig 2). While apical and basal regions may be affected to different degrees in COPD, the difference found here was not significant in the patient numbers examined. This may however be worthwhile to investigate in larger patient numbers. Similarly, it would be interesting to observe how the measurable perfusion inhomogeneity in diseased lungs reacts to hyperoxia, and doing so was one of the original goals of this study. However, we can only speculate whether the increase in heterogeneity detected at injection 2 on both days (Table 3), is due to the abovementioned effects of residual contrast agent, or whether it may be attributed to room air conditions as compared to pure O_2_. The latter at least is known to reduce hypoxic pulmonary vasoconstriction in COPD, thereby restoring a less heterogeneous perfusion pattern under hyperoxia [30].

Our study illustrates unexpected limitations of the quantitative evaluation of 4D lung perfusion MRI for short-term monitoring of lung function changes, in this case induced by hyperoxia. However, contrast agent-based lung perfusion measurement may still be evaluated qualitatively by visual scoring providing reliable functional information. Under the assumption that the observed problems are related to recirculation or tissue retention of contrast agent, it can be hypothesized that novel software or alternative approaches such as arterial spin labeling or Fourier Decomposition would be not affected by this limitation [31–33]. Further, the previous study [25] as well as the current are both limited by missing control subjects, in whom the experimental set-up was performed in a reverse manner on the same day. Finally, when planning a study that includes repeated contrast-agent based perfusion MRI acquisitions, the time interval should be appropriately chosen with regard to the patient cohort, e.g. two or more days for COPD patients.

In conclusion, automated software-based quantification of 4D perfusion datasets may be feasible for single measurements, but repeat contrast agent injections for monitoring short-term therapy effects may distort perfusion quantification by the currently available software methods. Also, in this context it is important not to rely on data known for healthy individuals, as the elimination half-life of the contrast agent may very well be longer in patients in whom renal impairment is a known comorbidity.

## Supporting information

S1 File Supporting information. Contains segmented lung volumes and Perfusion parameters after excluding large vessels.
(PDF)
