# Supplementary material for: Towards quantitative perfusion MRI of the lung in COPD: The problem of short-term repeatability
Source: PLoS One. 2018 Dec 10;13(12):e0208587. doi: 10.1371/journal.pone.0208587 (PMC6287948; doi:10.1371/journal.pone.0208587)
Supplement: S1 File — Contains segmented lung volumes and Perfusion parameters after excluding large vessels. (PDF) [file pone.0208587.s001.pdf]

Towards Quantitative Perfusion MRI of the Lung in COPD: The Problem of Short-Term Repeatability

Alvard Ter-Karapetyan, Simon M. F. Triphan, Bertram J. Jobst, Angela F. Anjorin, Julia Ley-Zaporozhan, Sebastian Ley, Oliver Sedlaczek, Jürgen Biederer, Hans-Ulrich Kauczor, Peter M. Jakob, Mark O. Wielpütz

Online Supplemental Information

SUPPLEMENTAL RESULTS

Segmented lung volumes

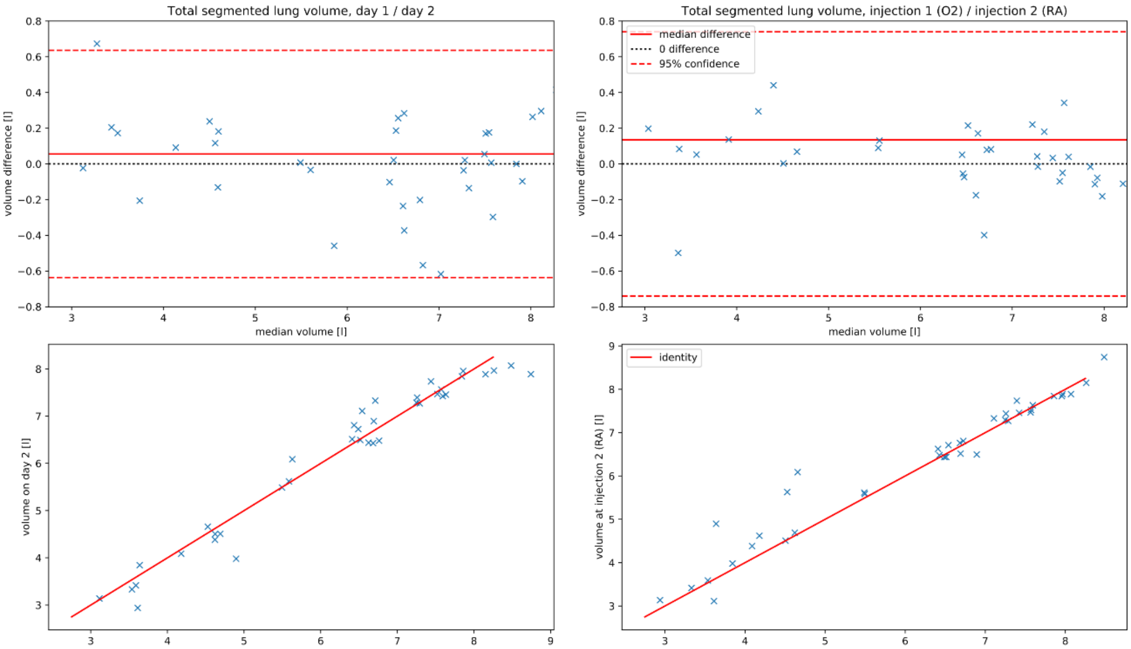

Figure A: Comparative plots of the segmented lung volumes detected in MRI measurements, relating volume on consecutive days as well as the different contrast agent injections.

The total lung volumes detected by segmentation of MR images is shown in figure S1. The found differences in lung volume were subjected to Wilcoxon signed-rank tests. On average, lung volumes were 0.16 l larger during injection 2 (p=0.07) on day 1 and 0.11 l (p=0.20) on day 2. Lung volumes were on average 0.03 l smaller on day 2 during injection 1 (p=0.75) and 0.03 l smaller during injection 2 (p=0.37). None of these differences were statistically significant with p<0.05.

Perfusion parameters excluding macroscopic vessels

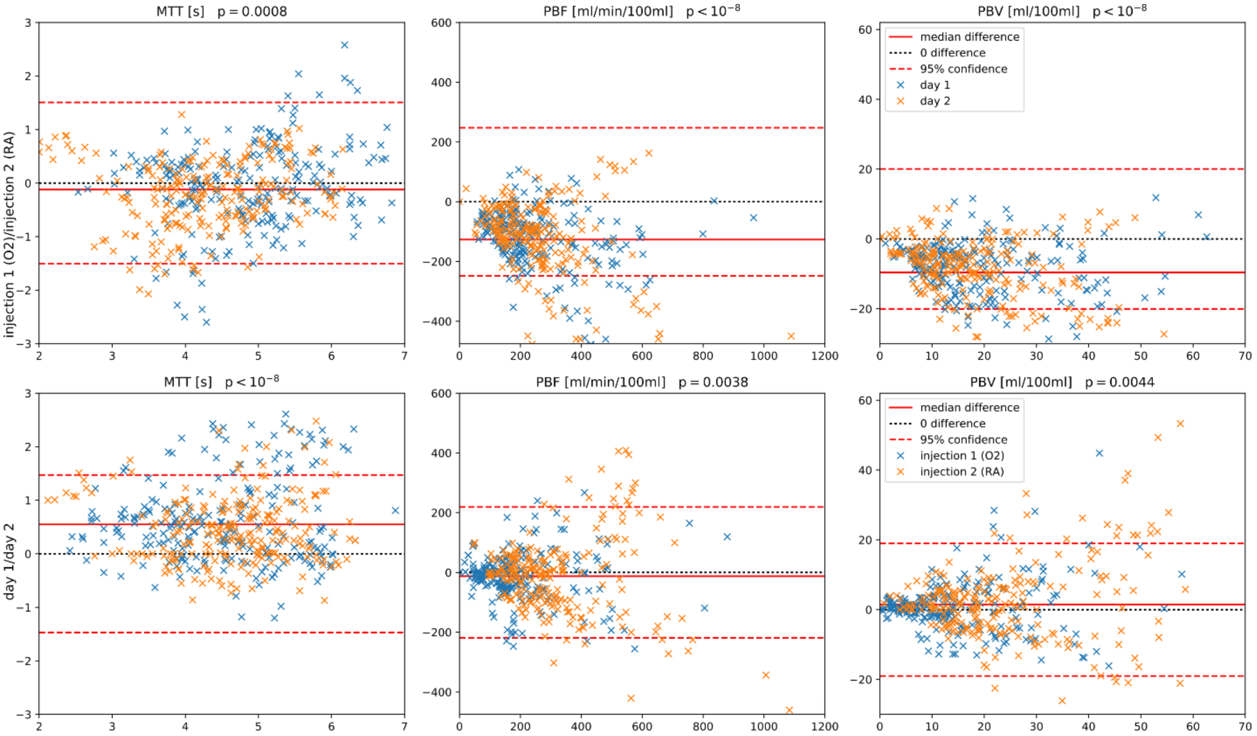

Figure B: In analogy to Figure 2 in the main manuscript, perfusion parameters taken from segmented lung regions with macroscopic vessels filtered out are compared. O2 = pure oxygen, RA = room air.

Figure S2 shows results equivalent to Figure 2 in the main manuscript, with the addition that a vascular suppression algorithm was applied, which subtracts major vasculature from the segmented lung volumes (1, 2). This has minimal effect on the statistical significance of results. Merely, the differences in PBV between day 1 and day 2 are closer to significance in this approach. Considering all segmented regions and both days, this yields significant differences between all parameters when comparing the differences between both injections and days. When examining the entire lungs, differences in PBF and PBV between injections are significant as well as differences in MTT between days

(at $p<10^{-4}$), just like without vascular suppression. The finding that calculated PBF and PBV are apparently higher with injection 2 (RA) is thus unchanged by vascular suppression.

SUPPLEMENTAL REFERENCES

1. Risse F, Kuder TA, Kauczor H-U, Semmler W, Fink C. Suppression of pulmonary vasculature in lung perfusion MRI using correlation analysis. *European Radiology* 2009; 19: 2569.
2. Kohlmann P, Strehlow J, Jobst B, Krass S, Kuhnigk J-M, Anjorin A, Sedlaczek O, Ley S, Kauczor H-U, Wielpütz MO. Automatic lung segmentation method for MRI-based lung perfusion studies of patients with chronic obstructive pulmonary disease. *International journal of computer assisted radiology and surgery* 2015; 10: 403-417.
